# The Oxyuris Vermicularis

**Published:** 1870-03

**Authors:** 


### The Oxyuris Vermicularis.

The editor of the Boston *Journal of Chemistry* in his issue for August, 1869, says:

We are pleased to notice that the views first presented through this *Journal,* regarding the place deposit of the ova of *Oxyuris vermicularis* in the alimentary canal, have reached the sanction of so distinguished an observer and physician as Prof. Venker, of Erlangen. He was the first to observe and recognize trichinia disease in the living subject, and may be regarded as the highest living authority upon all matters pertaining to parastic animals in man. In his recent report to the Congress of German Naturalists and Physicians, he states that "the ova of pin-worms *are only set free in the rectum, near the anus,* or after the worms have been discharged from the bowels." Any physician can verify this by taking a piece of silk or linen, and passing it across the external orifice of a child suffering from pin-worms, and examining the surface of the cloth with a lens of thirty or forty diameters. Hundreds of the minute white ova can be seen by the most inexperienced observer. As regards the use of lard or other fats as a remedy, externally applied, we can only say that its entire success is vouched for in numerous cases by physicians and others, and it is worthy of fair, extended trial. The parasite is exceedingly common, and very troublesome and vexatious; and, if the value of so simple and cheap a remedy is fully established, it will indeed prove a boon to thousands of suffering children and adults. The application should be thorough, and the surfaces of the sphincter muscle fully anointed.—*Reporter.*

